# Dynamic and scalable DNA-based information storage

**DOI:** 10.1038/s41467-020-16797-2

**Published:** 2020-06-12

**Authors:** Kevin N. Lin, Kevin Volkel, James M. Tuck, Albert J. Keung

**Affiliations:** 1 Department of Chemical and Biomolecular Engineering, North Carolina State University, Campus Box 7905, Raleigh, NC 27695-7905 USA; 2 Department of Electrical and Computer Engineering, North Carolina State University, Campus Box 7911, Raleigh, NC 27695-7911 USA

**Keywords:** Biotechnology, Synthetic biology, DNA computing and cryptography, Chemical engineering, Mathematics and computing

## Abstract

The physical architectures of information storage systems often dictate how information is encoded, databases are organized, and files are accessed. Here we show that a simple architecture comprised of a T7 promoter and a single-stranded overhang domain (ss-dsDNA), can unlock dynamic DNA-based information storage with powerful capabilities and advantages. The overhang provides a physical address for accessing specific DNA strands as well as implementing a range of in-storage file operations. It increases theoretical storage densities and capacities by expanding the encodable sequence space and simplifies the computational burden in designing sets of orthogonal file addresses. Meanwhile, the T7 promoter enables repeatable information access by transcribing information from DNA without destroying it. Furthermore, saturation mutagenesis around the T7 promoter and systematic analyses of environmental conditions reveal design criteria that can be used to optimize information access. This simple but powerful ss-dsDNA architecture lays the foundation for information storage with versatile capabilities.

## Abstract (web summary)

The physical architectures of information storage dictate how data is encoded, organised and accessed. Here the authors use DNA with a single-strand overhang as a physical address to access specific data and do in-storage file operations in a scalable and reusuable manner.

## Introduction

**Fig. 1:**
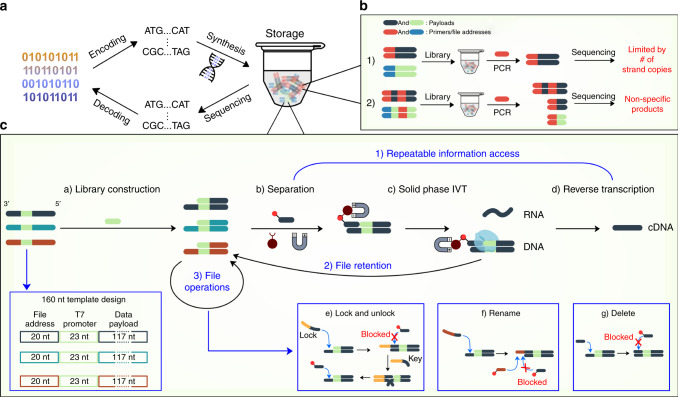
Molecular technologies unlock dynamic operations for DNA storage. **a** The generic framework for DNA-based storage systems includes encoding of digital information to nucleotide sequences, DNA synthesis and storage, DNA sequencing, and decoding the desired information. **b** Schematic of challenges faced by PCR-based file access. **c** Schematic of DORIS (Dynamic Operations and Reusable Information Storage). ss-dsDNA strands enable repeatable information access through non-PCR-based magnetic separation, in vitro transcription, reverse transcription, and the return of separated files to the database. Additionally, the overhangs of ss-dsDNAs enable in-storage file operations including lock, unlock, rename, and delete.

The creation of digital information is rapidly outpacing conventional storage technologies^1^. DNA may provide a timely technological leap due to its high storage density, longevity^2–4^, and energy efficiency^5^. A generic DNA-based information storage system is shown in Fig. 1a, where digital information is encoded into a series of DNA sequences, synthesized as a pool of DNA strands, read by DNA sequencing, and decoded back into an electronically compatible form. Recently, a growing body of work has focused on implementing and improving each of these four steps^6–11^; however, a relative dearth of research has explored technologies to access and manipulate desired subsets of data within storage databases^12,13^, especially dynamically. This is likely because DNA synthesis and sequencing are considerably slower processes than electronic writing and reading of data;^14^ thus, DNA would likely serve at the level of archival or cold storage where information would be infrequently accessed from a relatively static DNA database. Yet, an archival DNA database, just like electronic versions, need not be completely static and would benefit greatly from dynamic properties^15,16^. For example, in-storage file operations and computations and the ability to repeatedly access DNA databases would reduce DNA synthesis costs and abrogate the need to store multiple copies of archives. Therefore, implementation of dynamic properties would bring DNA-based storage systems one step closer to practical viability.

A practical system to dynamically access information from a DNA database should satisfy three criteria. It must be: (1) physically scalable to extreme capacities; (2) compatible with efficient and dense encodings; and (3) repeatedly accessible. Ideally, it would also be modifiable to some extent. While these criteria have not yet been achieved in aggregate, we were inspired by the creative use of molecular biology approaches in prior work to address some of these challenges. For example, polymerase chain reaction (PCR) is the predominant method for information access in DNA storage systems^7,12^ and is scalable, especially with some modifications^17^, while single-stranded DNA toeholds and strand displacement have been used for DNA computation^18–20^, DNA search^21^, detection^22^, and rewritable^12,23–25^ information storage. The challenge is that in their current form these technologies have inherent limitations and tradeoffs either in physical scalability, encoding density, or reusability. For example, while it is currently the most scalable and robust technique, PCR-based information access requires a portion of the database to be physically removed and amplified, with the number of data copies present dictating the number of times information can be accessed^6,26,27^. It also requires double-stranded DNA (dsDNA) templates to be melted in each cycle during which time primers can bind similar off-target sequences in the data payload regions, thus requiring encoding strategies that tradeoff reducing system densities and capacities to avoid these cross-interactions^7^ (Fig. 1b).

Here, we present a dynamic DNA-based storage system that satisfies these three criteria. It is inspired by work in the synthetic biology and molecular biology communities and by the way cells naturally access information in their genome. As described in Fig. 1c, we engineer an information storage system that has as its fundamental unit a double-stranded DNA with a single-stranded overhang (ss-dsDNA). A database of information would be comprised of many of such ss-dsDNA strands, with all strands that comprise a file having the same single-stranded overhang sequence or file address. The overhang also provides a handle with which a file can be separated as well as operated on in-storage. All strands have a T7 promoter enabling transcription of information into RNA, while the original ss-dsDNAs are retained and returned to the DNA database. This system can be created at scale, reduces off-target information access, facilitates computationally tractable design of orthogonal file addresses, increases information density and theoretical maximum capacity, enables repeatable information access with minimal strand copy number required, and supports multiple in-storage operations. This work demonstrates scalable dynamic information access and manipulations can be practical for DNA-based information storage. For convenience, we refer to this system collectively as DORIS (Dynamic Operations and Reusable Information Storage).

## Results

### ss-dsDNA strands can be efficiently created in one-pot

**Fig. 2:**
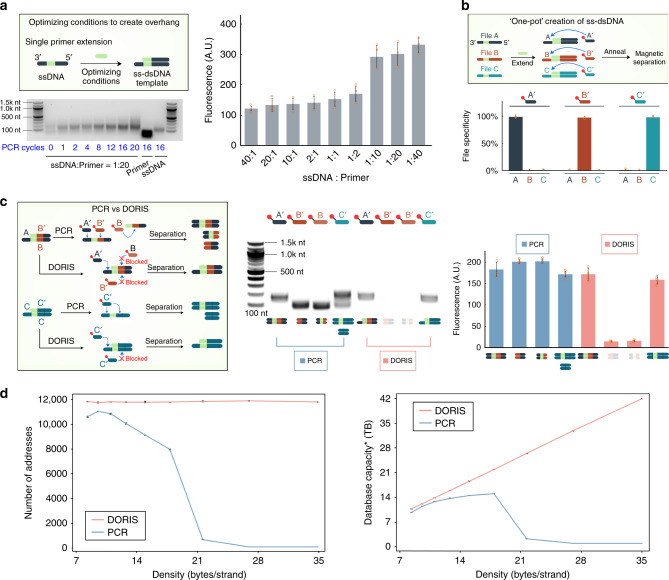
DORIS eliminates non-specific interactions and increases density and capacity limits. **a** Single primer extension created ss-dsDNAs. (Bottom) 4 cycles of PCR generated the optimal amount of 160 nt ss-dsDNAs while minimizing excess ssDNA production. (Right) DNA gel showed a marked increase in generation of ss-dsDNAs below 1:10 ssDNA:primer ratios. **b** Individual files can be separated from a three-file database created by a one-pot single primer extension. Each file was bound by its corresponding biotin-linked oligo, followed by a non-PCR-based separation using functionalized magnetic beads. File separation specificity is the percentage of the DNA separated by that is either file A, B, or C as measured by qPCR. **c** (Left) PCR but not DORIS will allow oligos to bind internal off-target sites and produce undesired products. (Middle) DNA gels and (Right) their quantified fluorescence (blue for PCR, pink for DORIS) showed that PCR-based access resulted in truncated and undesired amplicons whereas DORIS accessed only the desired strands. **d** (Left) Monte Carlo simulations estimated the number of oligos found that will not interact with each other or the data payload. 400,000 oligos were tested against different density encodings. The *x*-axis represents density (Eq. (4)), which is inversely related to the length of codewords used to store discrete one-byte data values. We evaluated codeword lengths of 12 through 4. For DORIS, the encoding density was not impacted because it need not guard against undesired binding between the oligos and data payloads. (Right) For PCR, the number of oligos that will not bind the data payload drops as strand density increases, which means that fewer files can be stored, leading to a lower overall system capacity. For DORIS, the availability of oligos is independent of encoding, and capacity therefore increases with denser encodings. Plotted values represent the arithmetic mean, and error bars represent the s.d., of three replicate file separations or simulations. Gel images are representative of three independent experiments measured by RT-QPCR. Source data are provided as a Source Data file. *Capacities may be limited by synthesis and sequencing limitations not accounted for here.

As future DNA databases would be comprised of upwards of 10^15^ distinct strands^17^, we first asked if ss-dsDNAs could be created in a high throughput and parallelized manner. We ordered 160 nucleotide (nt) single-stranded DNAs (ssDNA) with a common 23 nt sequence that was inset 20 nt from the 3’ end (Fig. 1c and 2a, Supplementary Table 1). This 23 nt sequence contained the T7 RNA polymerase promoter, but was also used to bind a common primer to fill-out and convert the ssDNA into a ss-dsDNA. This was achieved by several cycles of thermal annealing and DNA polymerase extension (e.g., PCR cycles but with only one primer). This resulted in ss-dsDNA strands with a 20 nt overhang (Fig. 2a, top). We optimized the ratio of ssDNA to primer, the number of cycles, along with other environmental parameters (Fig. 2a, Supplementary Fig. 1) to maximize the amount of ssDNA converted to ss-dsDNA. We found that decreasing the ssDNA:primer ratio past 1:10 led to a step change in the amount of ss-dsDNA produced as quantified by gel electrophoresis (Supplementary Fig. 1b). We decided to conservatively work with a 1:20 ssDNA:primer ratio. At that ratio we found that only 4 PCR cycles were needed to convert the ssDNA into ss-dsDNA, as seen by the upward shift in the DNA gel (Fig. 2a).

Next, we tested whether this method could be used to create 3 distinct ss-dsDNAs in one-pot reactions and if each ss-dsDNA could then be specifically separated from the mixture (Fig. 2b). We mixed 3 distinct ssDNAs “A”, “B”, and “C” together, added the common primer, and performed 4 PCR cycles to create the ss-dsDNAs (here referred to as files comprised of just one unique strand each). We then used biotin-linked 20 nt DNA oligos to bind each ss-dsDNA (i.e., each file, A, B, and C has a distinct overhang sequence or file address) and separated them out from the mixture using magnetic beads functionalized with streptavidin. Each of these oligos were able to specifically separate only their corresponding file without the other two (Fig. 2b, bottom, Eq. (1)). Importantly, this separation step could be performed at room temperature (25 °C) with only minimal gains observed at higher oligo annealing temperatures of 35 or 45 °C (Supplementary Fig. 2, Eq. (2)). The room temperature and isothermal nature of this step is useful for practical DNA storage systems and for reducing DNA degradation.

While 20 nt is a standard PCR primer length, we asked if the separation efficiency could be modulated by different overhang lengths and separation temperatures. We designed 5 ss-dsDNAs with 5–25 nt overhangs (Supplementary Fig. 3). We then separated each strand using its specific biotin-linked oligo at 15–55 °C. We observed enhanced separation efficiency for longer oligos (20mers and 25mers) and at lower temperatures (15 °C and 25 °C, Supplementary Fig. 3b). This was in agreement with a thermodynamic analysis using the Oligonucleotide Properties Calculator (Supplementary Fig. 3c, Methods, Eqs. (3)–(5))^28–30^.

### DORIS increases density and capacity limits

One potential advantage of room temperature separations of files is that the double-stranded portions of the ss-dsDNAs remain annealed together and may block undesired oligo binding to any similar sequences in the data payload regions. The data payload region is the majority of the sequence in the middle of ss-dsDNAs that contains the stored information. To test this hypothesis, we created two ss-dsDNAs (Fig. 2c). One ss-dsDNA had an overhang that bound oligo A’ and an internal binding site for oligo B’. We experimentally verified that by using DORIS, only oligo A’ but not oligo B’ could separate out the strand. For comparison, PCR-based systems melt dsDNAs in each cycle, allowing primers to bind off-target within the data payload. As expected, when PCR was used, both oligo A’ and oligo B’ bound, with oligo B’ producing undesired truncated products. The second strand we tested had an internal binding site and overhang that both were complementary to oligo C’. We showed that using DORIS, oligo C’ yielded only the full-length strand. In contrast, when using PCR, oligo C’ created both full length and truncated strands.

We next asked what implications this blocking property of DORIS had for DNA-based information storage. As databases increase in size, intuitively the likelihood for sequences identical to address sequences (either overhangs for DORIS or primer sites for PCR) appearing in data payload regions increases. With DORIS, this is not an issue as oligos are blocked from binding the dsDNA data payload regions. However, in PCR, primers do bind these data payload regions, so previous approaches have developed encoding algorithms that restrict primer sequences (addresses) from overlapping with any identical or similar sequence in the data payloads^11,12^, typically avoiding Hamming Distances within ~<6. This inherently reduces either the density with which databases can be encoded due to restrictions on data payload sequence space, or their capacity due to a reduction in the number of unique primer sequences that can be used. Density is the amount of information stored per nt (Eq. (6)), and it decreases as encoding restrictions are placed limiting what sequences can be used in the payload region (lower diversity sequence space), while capacity is the total amount of information that can be stored in a system (Eq. (7)) and is dependent on the number of addresses available as they dictate the number of files that can be stored.

To show these relationships quantitatively, it is currently intractable to analytically solve for or comprehensively compute the number of addresses available that do not interact with the data payload region, even for moderately sized databases. Therefore, we performed Monte Carlo simulations to estimate the total number of addresses and total capacities achievable. Address sequences were (PCR) or were not (DORIS) excluded if they appeared in the data payload regions of a database with 10^9^ distinct DNA strands (Fig. 2d, Methods). To simplify the analysis, we used computational codewords to encode the data payload region. Each codeword is a distinct nt sequence and holds one byte (B) of digital information. The data payload region can be made more information dense by reducing the size of the codewords so more codewords (and bytes) fit within each fixed-length strand. The tradeoff is that smaller codewords will also increase the sequence diversity of the strands (the number of possible distinct sequences per strand length) due to more codeword-codeword junctions per strand. This increases the chance of similar sequences appearing in the payload that conflict with address sequences.

The simulation assessed whether address sequences would conflict with any sequences in the payload. However, for DORIS, even if address sequences conflicted with the payload, these addresses were allowed. The simulation therefore showed that as the payload information density was increased by shrinking codeword length, the number of addresses available did not change for DORIS as no restrictions were placed on addresses other than that they were not allowed to be similar to other addresses (Fig. 2d, left, pink). Also as expected, as the payload information density increased, the database capacity increased monotonically as the number of file addresses remained the same as did the total number of strands per file (Fig. 2d, right, pink). In contrast, for PCR, addresses that appeared in any data payload sequence were excluded; the result was that increasing payload information density initially provided a minor benefit to overall capacity (Fig. 2d, right, blue) but eventually led to a catastrophic drop in capacity as the number of addresses that did not conflict with any payload sequence quickly dropped to zero (Fig. 2d, left, blue). While it is possible to increase the number of distinct strands per address (i.e., information per file) to make up for the loss of addresses, this would result in files too large to be sequenced and decoded in a single sequencing run^17^. It is also important to note that our simulations were based upon very conservative codeword densities and a database size of only 10^9^ DNA strands, while future storage systems are likely to exceed 10^12^ strands or greater. As database densities and DNA sequence spaces increase, the number of addresses available for PCR-based systems will drop even further while DORIS will be unaffected. Therefore, the theoretical capacity and density improvements DORIS provides could be orders of magnitude greater than what is estimated in our simulations. Furthermore, DORIS greatly simplifies address design; designing sets of orthogonal addresses for PCR-based systems that do not interact with data payload sequences will quickly become computationally intractable at large database sizes. In summary, a database comprised of ss-dsDNAs can be efficiently created in one-pot reactions, and ssDNA overhangs facilitate a non-PCR-based separation method that enhances address specificity and increases theoretical database densities and capacities.

### DORIS enables repeatable file access

**Fig. 3:**
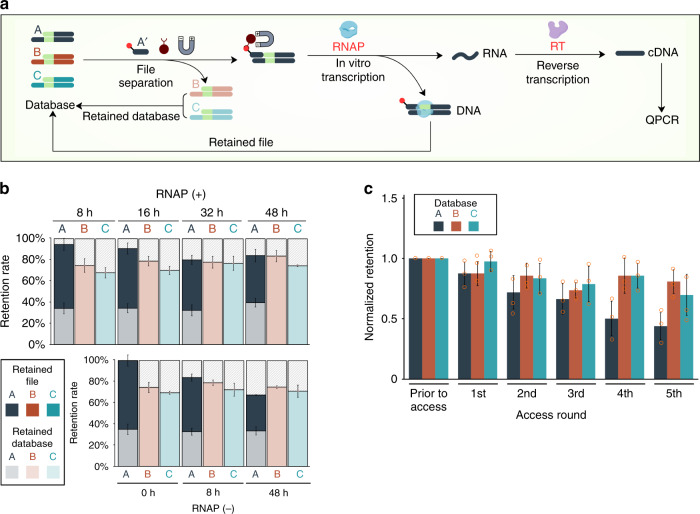
DORIS mimics natural transcription to repeatedly access information. **a** File A was separated using non-PCR-based magnetic separation while the database was recovered (Retained Database) (*n* = 3 for each condition). T7-based in vitro transcription was performed directly on the bead-immobilized file for up to 48 h to generate RNA. Reverse transcription converted the RNA to complementary DNA (cDNA) while the immobilized file A was released back into the database (Retained File) (*n* = 3 for each condition). **b** The amount of retained database (light shading) and retained file (dark shading) after file A was accessed by oligo A’ was measured by qPCR and plotted as a percentage of the original amount of each file that was in the database. The specificity of file access is evident by the absence of file B and C in the Retained File. The presence of T7 RNA polymerase (RNAP) did not affect the retention of file A. **c** File A was repeatedly accessed 5 times. The amounts of file A, B and C in the database were measured by qPCR and plotted as the amount of each file in the database after each run (*n* = 3 for each condition), normalized to the original amount of each file prior to the 1st access. Values represent the arithmetic mean. Error bars are s.d., *n* = the number of replicate file accesses. Source data are provided as a Source Data file.

A key requirement but major challenge for engineering dynamic properties into storage systems is the reusability of the system. In this work, we took inspiration from natural biological systems where information is repeatedly accessed from a single permanent copy of genomic DNA through the process of transcription. As shown in Fig. 3a, dynamic access in DORIS starts by physically separating out a file of interest (ss-dsDNAs sharing the same overhang address) using biotin-linked oligos and streptavidin-based magnetic separation, in vitro transcribing (IVT) the DNA to RNA^31^, returning the file to the database, and reverse-transcribing the RNA into cDNA for downstream analysis or sequencing.

We implemented this system with three distinct ss-dsDNAs (A, B, and C) collectively representing a three-file database, and we accessed file A with a biotinylated oligo A’ (Fig. 3b & Supplementary Fig. 4). We then measured the amounts and compositions of the “retained database” (light shading) and “retained file” (dark shading) by qPCR (Eq. (8)). The retained database had higher levels of files B and C compared to A, as some of the file A strands were removed in the magnetic separation. The retained file contained mostly file A strands, with minimal B or C. The best net total amount of file A recovered from the retained database and retained file was approximately 90% of what was originally in the database. The high retention rate of file A suggested that a file could be re-accessed multiple times. We tested this by repeatedly accessing file A five times, and measured the amounts and compositions of file A, B and C in the database after each access (Fig. 3c & Supplementary Fig. 4c). As expected, the overall amounts of file B and C were maintained at relatively stable levels in the database. Approximately 50% of file A strands remained after five accesses. The practical implications for DNA storage systems is that only 2 copies of each distinct sequence are needed in the initial database for every 5 times it is accessed (ignoring the effects of strand distributions). This is an improvement over PCR-based file access where small aliquots of the database are taken and amplified. In this case, one copy of each distinct sequence is needed for each access; furthermore, unlike in DORIS, all of the other database files will be similarly reduced in abundance even if they were not accessed. Thus, DORIS may extend the lifespan of DNA databases and allow for more frequent access for the same total mass of DNA synthesized.

We next asked how the IVT reaction might affect database stability, as it is performed at an elevated temperature of 37 °C and could degrade the ss-dsDNA. While the retained database is not exposed to the IVT, the accessed file is, and the amount of ss-dsDNA retained could be affected by the length of the IVT. Indeed, while the presence of RNA polymerase itself had no effect on the retained file, the length of IVT time did decrease the amount of retained file (Fig. 3b & Supplementary Fig. 4a). Interestingly, reannealing the retained file at 45 °C and allowing it to cool back to room temperature improved the retention rate, but longer IVT times still reduced overall file retention (Supplementary Fig. 4b). This suggests that some loss is due to the file strands unbinding from the bead-linked oligos or RNAs competing with ss-dsDNA, while some loss is due to DNA degradation. As a control to confirm that ss-dsDNA was not contaminating cDNA generated from the transcribed RNA, cDNA was obtained only when RNA polymerase was included in the IVT reaction (Supplementary Fig. 4d).

**Fig. 4:**
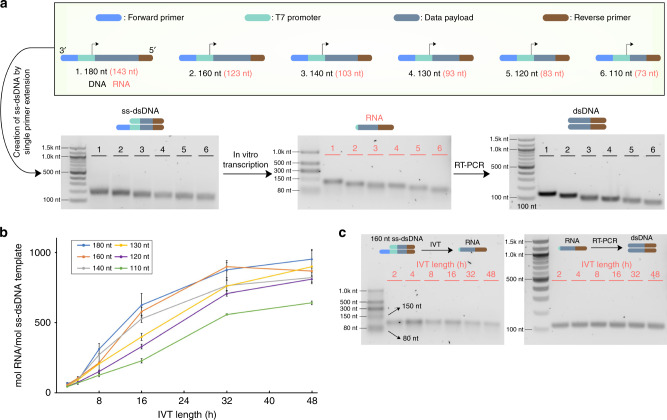
T7-based transcription generates uniformly sized products. **a** Six ssDNA oligos with different lengths were designed to generate six ss-dsDNA templates with lengths of 180 bp, 160 bp, 140 bp, 130 bp, 120 bp and 110 bp, respectively. Each ss-dsDNA comprised a consensus reverse primer binding sequence, T7 primer binding sequence, forward primer binding sequence, and a payload sequence with varying lengths. These ss-dsDNA templates were in vitro transcribed for 8 h, followed by RT-PCR. Product sizes were examined by agarose gel electrophoresis. **b** IVT time course for up to 48 h (*n* = 3 replicate IVT reactions for each condition). The amount of both RNA and DNA template molecules were measured by NanoDrop and plotted as their ratio. **c** Gel electrophoresis of RNA and dsDNA products after 2–48 h of IVT followed by RT-PCR. Plotted values represent the arithmetic mean, and error bars represent the s.d., of three independent IVT reactions. Gel images are representative for three independent experiments measured by RT-QPCR. Source data are provided as a Source Data file.

We next focused on assessing the quality and efficiency of the IVT. To check if RNA polymerase might be creating undesired truncated or elongated transcripts, we ordered a series of six ssDNAs with a range of lengths spanning 110–180 nt (Fig. 4a & Supplementary Fig. 5). These were converted into ss-dsDNA, transcribed into RNA, and reverse transcribed and amplified into dsDNA. Clear uniform bands were seen for the ss-dsDNA, RNA, and dsDNA. Increasing IVT time did increase the yield of RNA for all templates (Fig. 4b), although just 2 h was sufficient to obtain clear RNA bands (Fig. 4c), and IVT time did not affect the length of the RNA generated. In summary, information can be repeatedly accessed from ss-dsDNAs by oligo-based separation and IVT.

### Transcription can be tuned by promoter sequence

**Fig. 5:**
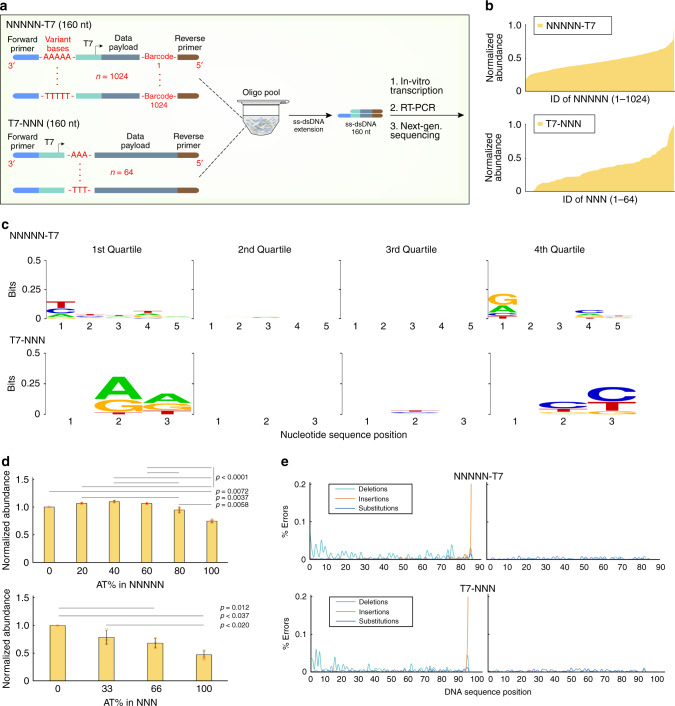
T7-based transcription efficiency can be controlled by surrounding sequences. **a** An oligo pool that had 1088 distinct sequences was designed to generate ss-dsDNA templates. The first 1024 sequences contained all possible combinations of nucleotides upstream of the promoter sequence (NNNNN-T7, where N is one of four DNA nucleotides), whereas the latter 64 sequences had all possible combinations of nucleotides downstream to the promoter region (T7-NNN). Each sequence contained a barcode to identify the sequence of the variant nucleotides. The template ss-dsDNAs were processed with IVT for 8 h, followed by RT-PCR and next-generation sequencing (*n* = 3 for each condition). **b** Transcription efficiencies of both sequence designs were plotted by normalizing the read count of each transcribed strand to its abundance in the original library. The data was organized from lowest to highest normalized abundance for both designs. **c** The sequences were further divided into four quartiles based upon normalized transcript abundance and analyzed by the WebLogo tool. **d** The normalized abundance of each sequence was organized by A/T percentage. *P* values between each group were calculated using One-Way ANOVA with Tukey–Kramer post-hoc and listed here for statistical significance. NNNNN-T7: *p* values less than 0.01 for comparisons between 0%–100%, 80%–100% and 20%–80%; *p* values less than 0.001 for comparisons between 20%–100%, 40%–80%, 40%–100%, 60%–80% and 60%–100%; T7-NNN, *p* values less than 0.05 for comparisons between 33%–100%, 0%–100% and 0%–66%. **e** The percent error for each DNA sequence position for the original synthesized database (left) and transcribed database (right). The error rate was calculated by dividing the number of errors of a given type occurring at a nucleotide position by the total number of reads for that sequence (Supplementary Method). Plotted values represent the arithmetic mean, and error bars represent the s.d., of three independent IVT-RT-PCR-NGS samples. Source data are provided as a Source Data file.

Recent works on molecular information storage have demonstrated the utility of storing additional information in the composition of mixtures of distinct molecules, including DNA^32,33^. As the information accessed by DORIS relies on the T7 RNA polymerase, and there is evidence that T7 promoter variants can affect transcription efficiency^34–38^, we asked whether the yield of T7-based transcription could be modulated by specific nucleotide sequences around the T7-promoter region while keeping the promoter itself constant to allow for one-pot ss-dsDNA generation (Fig. 2a, b). To comprehensively address this question, we designed and ordered 1088 distinct 160 nt strands as an oligo pool. The first 1024 strands contained all possible 5 nt variant sequences upstream to the promoter sequence (NNNNN-Promoter, N is each of the four nucleotides), and the latter 64 sequences were all 3 nt variant sequences downstream of the promoter (Promoter-NNN, Fig. 5a). As the NNNNN nucleotides were located in the ssDNA overhang, we also asked if this region being single stranded versus double stranded had any impact on relative transcriptional efficiencies. We first created ss-dsDNA by primer extension and dsDNA by PCR of the ssDNA oligo pool. Both ss-dsDNA and dsDNA databases were processed with IVT at 37 °C for 8 h, followed by RT-PCR and next-generation sequencing. Short barcodes were designed in the payload region to identify which promoter variant each sequenced transcript was derived from.

The abundance of each distinct transcript sequence was normalized to its abundance in the original ss-dsDNA (Fig. 5b) or dsDNA (Supplementary Fig. 6a) database (Eq. (9)). A broad and nearly continuous range of normalized abundances was obtained, indicating that this approach could be harnessed to create complex compositional mixtures of DNA in the future. To determine if there may be simple design principles that described promoter efficiency, we segmented the 1088 sequences into quartiles based on transcript abundance and imported the data into the WebLogo tool^39^. We found that G or A at the 5th position directly upstream and C or T at the 3rd position directly downstream of the T7 promoter generally resulted in the highest RNA abundances (Fig. 5c). Segmenting the data by A/T content showed that there was a slight preference for ~50% A/T content upstream of the T7 promoter and preference for overall low A/T content downstream of the T7 promoter (Fig. 5d).

This next-generation sequencing experiment also provided confidence that DORIS is scalable to large and complex ss-dsDNA pools. Furthermore, error analysis of the sequencing reads indicated no systematic deletions, truncations, or substitutions, and overall error levels were well below those already present from DNA synthesis (Fig. 5e).

### DORIS enables in-storage file operations

**Fig. 6:**
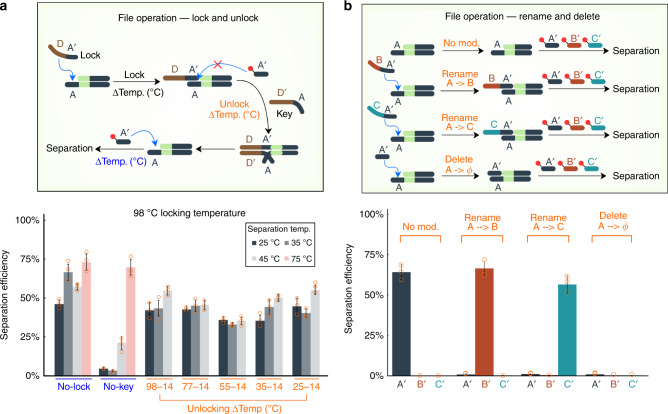
Toeholds enable in-storage file operations. **a** (Top) Schematic of locking and unlocking in-storage file operations. (Bottom) Attempts to access file A by DORIS without locking (No-Lock), with locking but without a key (No-Key), or with locking and key added at different temperatures (orange) (*n* = 3 for each condition). The lock was added at 98 °C. The key was added at different temperatures (orange) and then cooled to 14 °C (*n* = 3 for each condition). Oligo A’ was added at different access temperatures of 25, 35, 45, or 75 °C for 2 min, followed by a temperature drop of 1 °C/min to 25 °C (*n* = 3 for each condition). Separation efficiency is the amount of file A recovered relative to its original quantity, as measured by qPCR. **b** (Top) Schematic of rename and delete operations. File A was modified by renaming or deleting oligos. (Bottom) The completion of each operation was tested by measuring how much of the file was separated by each individual oligo: A’, B’, or C’. Separation efficiency is the amount of file A separated relative to its original amount in the database, as measured by qPCR. No Mod (No file modification/operation). Plotted values represent the arithmetic mean, and error bars represent the s.d., of three independent replicate file operations/separations. Source data are provided as a Source Data file.

Many inorganic information storage systems, even cold storage archives, maintain the ability to dynamically manipulate files. Similar capabilities in DNA-based systems would significantly increase their value and competitiveness. ssDNA overhangs have previously been used to execute computations in the context of toehold switches^40–43^, and we therefore hypothesized they could be used to implement in-storage file operations. As a proof-of-principle, we implemented locking, unlocking, renaming, and deleting files and showed these operations could be performed at room temperature (Fig. 6).

We started with the three-file database and tested the ability of a biotin-linked oligo A’ to bind and separate file A at a range of temperatures from 25 to 75 °C (Fig. 6a, bottom, no lock). Roughly 50% of file A strands were successfully separated from the database. To lock file A, we separated file A from the three-file database and mixed in a long 50 nt ssDNA (lock) that had a 20 nt complementary sequence to the ssDNA overhang of file A. With the lock in place, oligo A’ was no longer able to separate the file except at higher temperatures above 45 °C (Fig. 6a, bottom, no-key), presumably because the lock was melted from the overhang, allowing for oligo A’ to compete to bind the overhang. To unlock the file, we added the key that was a 50 nt ssDNA fully complementary to the lock. We tested different unlocking temperatures and found the key was able to remove the lock at room temperature with the same efficiency as at higher temperatures. This is likely due to the long 30 nt toehold presented by the lock, allowing the key to unzip the lock from file A. We also optimized the relative molar ratios (file A: lock: key: oligo A’ = 1: 10: 10: 15) to minimize off-target separation and ensure proper locking. We did observe that the temperature at which the lock was added influenced the fidelity of the locking process. At 98 °C, the locking process worked well. When the lock was added at 25 °C, there was leaky separation even when no key was added (Supplementary Fig. 7). This may be due to secondary structures preventing some file A strands from hybridizing with locks at low temperatures. Fortunately, locking at 45 °C had reasonable performance, thus avoiding the need to elevate the system to 98 °C. In the context of a future DNA storage system, files could first be separated then locked at an elevated temperature, then returned to the database, thus avoiding exposure of the entire database to elevated temperatures. The entire process could otherwise be performed at room temperature.

We also implemented file renaming and deletion. To rename a file with address A to have address B, we mixed file A with a 40 nt ssDNA that binds to A, with the resultant overhang being address B (Fig. 6b). We added all components at similar ratios to the locking process (file: renaming oligo: accessing oligo = 1: 10: 15) and the renaming oligo was added at 45 °C. We then tested how many file strands each oligo A’, B’, or C’ could separate and found that the renaming process completely blocked oligos A’ or C’ from separating out the file (Fig. 6b, bottom). Only oligo B’ was able to separate the file suggesting that almost all strands were successfully renamed from A to B. Similarly, we successfully renamed file A to C. Based on the ability of oligos to rename files with near 100% completion, we hypothesized and indeed found that a short 20 nt oligo fully complementary to A could be used to completely block the overhang of file A and essentially delete it from the database (Fig. 6b, bottom). A file could also simply be extracted from a database to delete it as well. However, this alternative form of blocking-based deletion suggests one way to ensure any leftover file strands that were not completely extracted would not be spuriously accessed in the future.

## Discussion

As DNA-based information storage systems approach practical implementation^44,45^, scalable molecular advances are needed to dynamically access information from them. DORIS represents a proof of principle framework for how inclusion of a few simple innovations can fundamentally shift the physical and encoding architectures of a system. In this case, ss-dsDNA strands drive multiple powerful capabilities for DNA storage: (1) it provides a physical handle for files and allows files to be accessed isothermally; (2) it increases the theoretical information density and capacity of DNA storage by inhibiting non-specific binding within data payloads and reducing the stringency and overhead of encoding; (3) it eliminates intractable computational challenges associated with designing orthogonal sets of address sequences; (4) it enables repeatable file access via in vitro transcription; (5) it provides control of relative strand abundances; and (6) it makes possible in-storage file operations. We envision other innovative architectures and capabilities may be on the horizon given rapid advances in DNA origami^46^, molecular handles^47–49^, and molecular manipulations developed in fields such as synthetic biology^50^.

Beyond the specific capabilities enumerated above, one of the greatest benefits we envision DORIS providing is compatibility with future miniaturized and automated devices^44,45^. In particular, DORIS can operate isothermally and function at or close to room temperature for all steps. This has potential advantages for maintaining DNA integrity and database stability while also simplifying the design of future automated DNA storage devices. In addition, a single DNA database sample can be reused, extending the lifespan of storage systems. It is also intriguing to consider what other types of in-storage operations like lock & unlock can be implemented to offer unique unforeseen capabilities in the future. All of these features lend DORIS to be easily translated to systems with automated fluid handling and magnetic actuation.

DORIS is also a fundamentally scalable system. The creation of ss-dsDNA strands is simple and high throughput, it is compatible with existing file system architectures including hierarchical addresses^11,17^, and it facilitates scaling of capacity. While the need to include the T7 promoter in every strand does occupy valuable data payload space, it is a worthwhile tradeoff: the T7 promoter decreases data density and capacity in a linear fashion, yet it more than compensates by simultaneously improving both metrics exponentially by allowing many sequences to appear in the data payload that normally would have to be avoided in PCR-based systems (or conversely by allowing the full set of mutually non-conflicting addresses to be used)^11,17^. It is also important to note that DORIS may help solve scalability issues with the encoding process as well: cross-comparing all address sequences with all data payload sequences is computationally intractable, but the need to do this is eliminated with DORIS as addresses will not physically interact with data payload sequences. Future work may assess how DORIS and other physical innovations may alter and reduce the stringency of encoding and error correction algorithms and subsequently benefit system density and capacity. Furthermore, with the insights into the impact of the sequence space surrounding the T7 promoter on transcriptional yield, an additional layer of information could be stored in the quantitative composition of DNA mixtures.

Of course, as with all information storage systems, there are challenges and questions regarding the efficiency and accuracy of each technology that will be important to address prior to commercial implementation. For example, future work might assess how each step of DORIS performs in the context of increasingly diverse and dense pools of strands, both in terms of efficiency and information retrieval error rates. In particular, new materials, RNA polymerase enzymes, and the optimization of reaction conditions could improve DNA recovery percentages to drive DORIS towards a fully reusable system. Devoting resources and attention to such optimizations need to be balanced with the fact that the field of molecular information storage is nascent and that there are likely a wide range of new capabilities and physical innovations that could be explored and introduced into the field.

Finally, we believe this work motivates a merging of work in the fields of DNA computation, synthetic biology, and DNA storage. In-storage computation and file operations could increase the application space of DNA storage, or identify cutting-edge applications areas, such as in the highly parallel processing of extreme levels of information (e.g., medical, genomic, and financial data). DORIS complements and harnesses the benefits of prior work while providing a feasible path towards future systems with advanced capabilities.

## Methods

### Creation of ss-dsDNA strands

ss-dsDNA strands were created by filling in ssDNA templates (IDT DNA) with primer TCTGCTCTGCACTCGTAATAC (Eton Bioscience) at a ratio of 1:40 using 0.5 µL of Q5 High-Fidelity DNA Polymerase (NEB, M0491S) in a 50 µL reaction containing 1x Q5 polymerase reaction buffer (NEB, B9072S) and 2.5 mM each of dATP (NEB, N0440S), dCTP (NEB, N0441S), dGTP (NEB, N0442S), dTTP (NEB, N0443S). The reaction conditions were 98 °C for 30 s and then 4 cycles of: 98 °C for 10 s, 53 °C (1 °C s^−1^ temperature drop) for 20 s, 72 °C for 10 s, with a final 72 °C extension step for 2 min. ss-dsDNA strands were purified using AMPure XP beads (Beckman Coulter, A63881) and eluted in 20 μL of water.

### File separations

Oligos were purchased with a 5′ biotin modification (Eton Bioscience, Supplementary Table 1). ss-dsDNA strands were diluted to 10^11^ strands and mixed with biotinylated oligos at a ratio of 1:40 in a 50 µL reaction containing 2 mM MgCl_2_ (Invitrogen, Y02016) and 50 mM KCl (NEB, M0491S). Oligo annealing conditions were 45 °C for 2 min, followed by a temperature drop at 1 °C/min to 14 °C. Streptavidin magnetic beads (NEB, S1420S) were prewashed using high salt buffer containing 20 mM Tris-HCl, 2 M NaCl and 2 mM EDTA pH 8 and incubated with ss-dsDNA strands at room temperature for 30 min. The retained database was recovered by collecting the supernatant of the separation. The beads were washed with 100 µL of high salt buffer and used directly in the in vitro transcription reaction. After transcription, the beads with the bound files were washed twice with 100 µL of low salt buffer containing 20 mM Tris-HCl, 0.15 M NaCl and 2 mM EDTA pH 8 and subsequently eluted with 95% formamide (Sigma, F9037) in water. The quality and quantity of the DNA in the retained database and file were measured by quantitative real-time PCR (Bio-Rad).

### In vitro transcription

Immobilized ss-dsDNA strands bound on the magnetic beads were mixed with 30 µL of in vitro transcription buffer (NEB, E2050) containing 2 µL of T7 RNA Polymerase Mix and ATP, TTP, CTP, GTP, each at 6.6 mM. The mixture was incubated at 37 °C for 8, 16, 32, and 48 h, followed by a reannealing process where the temperature was reduced to 14 °C at 1 °C/min to enhance the retention of ss-dsDNA on the beads. The newly generated RNA transcripts were separated from the streptavidin magnetic beads and their quantity measured using the Qubit RNA HS Assay Kit (Thermo Fisher, Q32852) and Fragment Analyzer Small RNA Kit (Agilent Technologies Inc., DNF-470-0275).

### Gel electrophoresis for DNA

Agarose-based DNA gels were made by mixing and microwaving 100 mL of 1x LAB buffer containing 10 mM Lithium acetate dihydrate pH 6.5 (VWR, AAAA17921-0B) and 10 mM Boric acid (VWR, 97061–974) with 1.5 mg of molecular biology grade agarose (GeneMate, 490000–002). 0.1x SYBR Safe DNA Gel Stain was added to visualize DNA (Invitrogen, S33102). DNA samples and ladder (NEB, N3231S) were loaded with 1x DNA loading dye containing 10 mM EDTA, 3.3 mM Tris-HCl (pH 8.0), 0.08% SDS and 0.02% Dye 1 and 0.0008% Dye 2 (NEB, B7024S). Electrophoresis was performed with 1x LAB buffer in a Thermo Scientific Mini Gel Electrophoresis System (Fisher Scientific, 09–528–110B) at a voltage gradient of 25 V/cm for 20 min.

### Gel electrophoresis for RNA

All equipment was cleaned by 10% bleach (VWR, 951384) and RNaseZap (Fisher Scientific, AM9780) to minimize nuclease contamination, particularly ribonuclease (RNase) contamination. The following procedures were performed in a PCR workstation with sterile pipetting equipment to further reduce ribonuclease contamination. Agarose-based RNA gels were cast by mixing and microwaving 100 mL of 1x TAE buffer containing 0.04 M Tris-Acetate and 0.001 M EDTA pH 8.0 with 1.5 mg of molecular biology grade agarose (GeneMate, 490000–002). 0.1x of SYBR Safe Gel Stain (Invitrogen, S33102) was added to visualize the RNA. RNA samples were treated with 2 units DNase I (NEB, M0303S) and incubated at 37 °C for 10 min, followed by a purification process using Monarch RNA Cleanup Kit (NEB, T2030S). The purified samples and RNA ladder (NEB, N0364S) were mixed with 1x RNA loading dye containing 47.5% Formamide, 0.01% SDS, 0.01% bromophenol blue, 0.005% Xylene Cyanol and 0.5 mM EDTA (NEB, B0363S). The mixtures were heated up at 65 C for 10 min, followed by immediate cooling on ice for 5 min. RNA electrophoresis was performed at a voltage gradient of 15 V/cm for 45 min.

### Gel imaging

Fluorescence imaging of both DNA and RNA gel samples was performed with a Li-Cor Odyssey® Fc Imaging System and the fluorescence intensity was quantified using FIJI software.

### Reverse transcription

First-strand synthesis was generated by mixing 5 µL of RNA with 500 nM of reverse primer in a 20 µL reverse transcription reaction (Bio-Rad, 1708897) containing 4 µL of reaction supermix, 2 µL of GSP enhancer solution, and 1 µL of reverse transcriptase. The mixture was incubated at 42 °C for 30 or 60 min, followed by a deactivation of the reverse transcriptase at 85 °C for 5 min. To generate ample product for gel electrophoresis analyses, the resultant cDNA was diluted 100-fold, and 1 µL was used as the template in a PCR amplification containing 0.5 µL of Q5 High-Fidelity DNA Polymerase (NEB, M0491S), 1x Q5 polymerase reaction buffer (NEB, B9072S), 0.5 uM of forward and reverse primer, 2.5 mM each of dATP (NEB, N0440S), dCTP (NEB, N0441S), dGTP (NEB, N0442S), dTTP (NEB, N0443S) in a 50 µL total reaction volume. The amplification conditions were 98 °C for 30 s and then 25 cycles of: 98 °C for 10 s, 55 °C for 20 s, 72 °C for 10 s with a final 72 °C extension step for 2 min. The products were assayed by gel electrophoresis and their concentrations were measured by Fragment Analyzer HS NGS Fragment Kit (Agilent Technologies Inc., DNF-474-0500).

### Locking and unlocking

Lock and key strands were purchased from Eton Biosciences. To lock the file, purified ss-dsDNA strands were mixed with lock strands at a molar ratio of 1:10 in a 25 µL reaction containing 2 mM MgCl_2_ and 50 mM KCl. The mixture was annealed to 98 °C, 45 °C or 25 °C for 2 min, followed by a temperature drop at 1 °C/min to 14 °C. To unlock the file, key strands were added into the locked file mixture at a molar ratio of 10:1 to the original ss-dsDNA strand amount. The mixtures were annealed to 98, 77, 55, 35, or 25 °C for 2 min, followed by a temperature drop at 1 °C/min to 14 °C. To access the unlocked strands, file-specific biotin-modified oligos were added into the mixture at a ratio of 15:1 to the original ss-dsDNA strand amount supplemented with additional MgCl_2_ and KCl to a final concentration of 2 mM and 50 mM, respectively, in a 30 µL reaction.

### Renaming and deleting

ss-dsDNA strands were mixed with renaming or deleting oligos at a ratio of 1:20 in a 25 µL reaction containing 2 mM MgCl_2_ and 50 mM KCl. The mixture was heated to 35 °C for 2 min, followed by a temperature drop at 1 °C/min to 14 °C. To delete the file, oligos were mixed with purified target file strands at a ratio of 1:20.

### Real-time PCR (qPCR)

qPCR was performed in a 6 μL, 384-well plate format using SsoAdvanced Universal SYBR Green Supermix (BioRad, 1725270). The amplification conditions were 95 °C for 2 min and then 50 cycles of: 95 °C for 15 s, 53 °C for 20 s, and 60 °C for 20 s. Quantities were interpolated from the linear ranges of standard curves performed on the same qPCR plate.

### Poly A tailing and template elongation

The NNN sequences in Fig. 5 and Supplementary Fig. 6 are captured in the cDNA samples. However, they cannot be immediately amplified in preparation for next-generation sequencing as a common PCR primer pair is not available. Therefore, cDNA was A-tailed with terminal transferase under the following reaction conditions: 5.0 uL of 10x TdT buffer, 5.0 uL of 2.5 mM CoCl_2_ solution provided with the buffer, 5.0 pmols of the amplified cDNA samples, 0.5 uL of 10 mM dATP (NEB, N0440S), and 0.5 uL of terminal transferase (20 units/uL) (NEB, M0315S) in a 50 uL total reaction volume. The mixture was incubated at 37 °C for 30 min, and then 70 °C for 10 min to deactivate the enzyme. The A-tailed samples were further amplified using the primers provided in Supplementary Table 1 to extend the length of each sequence for optimal next-generation sequencing. The PCR reaction used the following recipe: 0.5 µL of Q5 High-Fidelity DNA Polymerase (NEB, M0491S), 1x Q5 polymerase reaction buffer (NEB, B9072S), 0.5uM of forward and reverse primer, 2.5 mM each of dATP (NEB, N0440S), dCTP (NEB, N0441S), dGTP (NEB, N0442S) and dTTP (NEB, N0443S) in a 50 µL total reaction volume. The amplification conditions were 98 °C for 30 s, 25 cycles of: 98 °C for 10 s, 55 °C for 20 s, 72 °C for 10 s, with a final 72 °C extension step for 2 min. The products were assayed by gel electrophoresis.

### Next-generation sequencing

Amplicons were purified with AMPure XP beads (Beckman Coulter, A63881) according to the TruSeq Nano protocol (Illumina, 20015965). The quality and band sizes of libraries were assessed using the High Sensitivity NGS Fragment Analysis Kit (Advanced Analytical, DNF-474) on the 12 capillary Fragment Analyzer (Agilent Technologies Inc.). The prepared samples were submitted to Genewiz Inc. for Illumina-based next-generation sequencing (Amplicon-EZ). Ligation of Illunima sequencing adapters to the prepared samples was performed by Genewiz Inc. Next-generation sequencing data were analyzed as described in Supplementary Fig. 8.

### File specificity

In Fig. 2, we calculated file specificity by the following equation

1 $${\mathrm{File}}\,{\mathrm{Specificity}}\left( {\mathrm{\% }} \right) = \frac{{{\mathrm{Amount}}\,{\mathrm{of}}\,{\mathrm{strands}}\,{\mathrm{of}}\,{\mathrm{a}}\,{\mathrm{specific}}\,{\mathrm{file}}\,{\mathrm{in}}\,{\mathrm{a}}\,{\mathrm{solution}}}}{{{\mathrm{Total}}\,{\mathrm{number}}\,{\mathrm{of}}\,{\mathrm{strands}}\,{\mathrm{in}}\,{\mathrm{the}}\,{\mathrm{same}}\,{\mathrm{solution}}}}$$

### Separation efficiency

In Fig. 3, Supplementary Figs. 2, 3, and 7 we calculated the separation efficiency by the following equation.

2 $${\mathrm{Separation}}\,{\mathrm{Efficiency}}\left( {\mathrm{\% }} \right) = \frac{{{\mathrm{A}},{\mathrm{B}},{\mathrm{or}}\,{\mathrm{C}}\,{\mathrm{in}}\,{\mathrm{the}}\,{\mathrm{separated}}\,{\mathrm{sample}}\,{\mathrm{eluted}}\,{\mathrm{from}}\,{\mathrm{bead}}}}{{{\mathrm{Amount}}\,{\mathrm{of}}\,{\mathrm{A}},{\mathrm{B}},{\mathrm{or}}\,{\mathrm{C}}\,{\mathrm{in}}\,{\mathrm{database}}\,{\mathrm{before}}\,{\mathrm{separation}}}}$$

### Theoretical thermodynamic calculations

To theoretically estimate the fraction of bound oligos of various lengths and at different temperatures (Supplementary Fig. 3c), we calculated the equilibrium constants at each condition:

3 $$K = {\mathrm{exp}}\left( { - \frac{{\Delta \mathrm{G}^0}}{{\mathrm{RT}}}} \right)$$

Where ∆G^0^ is the change in Gibbs Free Energy at standard conditions (25 °C, pH = 7); R is the gas constant and T is the reaction temperature. The Gibbs Free Energy for each oligo was obtained using the Oligonucleotide Properties Calculator^28–30^. The equilibrium constant at each condition was equated to

4 $$K = \frac{{[Oligo - ssdsDNA]}}{{\left[ {ssdsDNA} \right] * \left[ {Oligo} \right]}}$$

with:

5 $$\frac{{\left[ {Oligo - ssdsDNA} \right]}}{{[ssdsDNA]}} = K * \left[ {Oligo} \right]$$

representing the fraction of strands separated out to the total original amount of ss-dsDNA strands. This amount, expressed as a percentage, is referred to as the separation efficiency.

### Density and capacity calculation

Experimental work was performed using the oligos listed in Supplementary Table 1. Simulation densities were measured by calculating the number of bytes in a 160 nt data payload with 5 codewords used for the strand index^11^, with the codeword length given as L:

6 $${\mathrm{Density}} = \left( {160 - 5 * L} \right)/L$$

The size of the index was chosen to accommodate 10^9^ strands.

Capacity: For each density and corresponding number of oligos, system capacity was calculated assuming 10^9^ strands per file, which roughly corresponds to the number of strands that can be sequenced at a time in next-generation sequencing. We further assumed that each strand occurred 10 times in replicate.

7 $${\mathrm{Capacity}} = 10^9 * \left( {{\mathrm{Number}}\,{\mathrm{of}}\,{\mathrm{Primers}}} \right) * {\mathrm{Density}}/10$$

Note, these capacity calculations were based on the number of oligos found in our search (Supplementary Fig. 9), not the total number that may be available if we searched the entire space of all possible 20 nt oligos. Searching for more oligos will result in greater system capacity, but searching the entire space is intractable using our current approach.

### Retention rate

In Fig. 6 and Supplementary Fig. 4, we calculated the retention rate of strands A, B, and C in the retained database or retained file. For clarity, all retention rate calculations were based on the molar amounts of DNA using this equation.

8 $${\mathrm{Retention}}\,{\mathrm{Rate}}\left( {\mathrm{\% }} \right) = \frac{{{\mathrm{Amount}}\,{\mathrm{of}}\,{\mathrm{A}},{\mathrm{B}},{\mathrm{or}}\,{\mathrm{C}}\,{\mathrm{in}}\,{\mathrm{retained}}\,{\mathrm{database}}\,{\mathrm{or}}\,{\mathrm{retained}}\,{\mathrm{file}}}}{{{\mathrm{Starting}}\,{\mathrm{amount}}\,{\mathrm{of}}\,{\mathrm{A}},{\mathrm{B}},{\mathrm{or}}\,{\mathrm{C}}\,{\mathrm{prior}}\,{\mathrm{to}}\,{\mathrm{separation}}}}$$

### Normalized abundance

In Fig. 5 and Supplementary Fig. 6, we calculated the normalized abundance of strands using the equation

9 $${\mathrm{Normalized}}\,{\mathrm{Abundance}} = \frac{{{\mathrm{Reads}}\,{\mathrm{of}}\,{\mathrm{a}}\,{\mathrm{strand}}\left( {{\mathrm{after}}\,{\mathrm{IVT}} - {\mathrm{RT}} - {\mathrm{PCR}} - {\mathrm{NGS}}} \right)}}{{{\mathrm{Reads}}\,{\mathrm{of}}\,{\mathrm{the}}\,{\mathrm{same}}\,{\mathrm{strand}}\,{\mathrm{in}}\,{\mathrm{the}}\,{\mathrm{original}}\,{\mathrm{database}}\,{\mathrm{sample}}}}$$

### Reporting summary

Further information on research design is available in the Nature Research Reporting Summary linked to this article.

## Supplementary information

Supplementary Information

Reporting Summary

## Data Availability

The Source Data for the figures presented in this manuscript and the Supplementary Information are available in the Source Data file and at https://github.com/jamesmtuck/DORIS/releases/download/v1.0/Supplemental.Data.File.1.zip. All other data are available upon reasonable request.

## Source data

Source Data

## Code availability

The software algorithms we developed to perform the reported analyses are available at https://github.com/jamesmtuck/DORIS under a permissive open source license with instructions for installation. We implemented code in python using many standard open source packages, including biopython, primer3, numpy, scipy, pandas, and others. These dependences are documented in the form of a python requirements.txt file that guides installation of additional dependent software packages. A docker file is available to make setup on a wide variety of systems easier.
