# Supplementary material for: Dynamic and scalable DNA-based information storage
Source: Nat Commun. 2020 Jun 12;11:2981. doi: 10.1038/s41467-020-16797-2 (PMC7293219; doi:10.1038/s41467-020-16797-2)
Supplement: Supplementary file 1 — Supplementary Information [file 41467_2020_16797_MOESM1_ESM.pdf]

SUPPLEMENTARY INFORMATION

Dynamic and scalable DNA-based information storage. Lin et al

Supplementary Figures

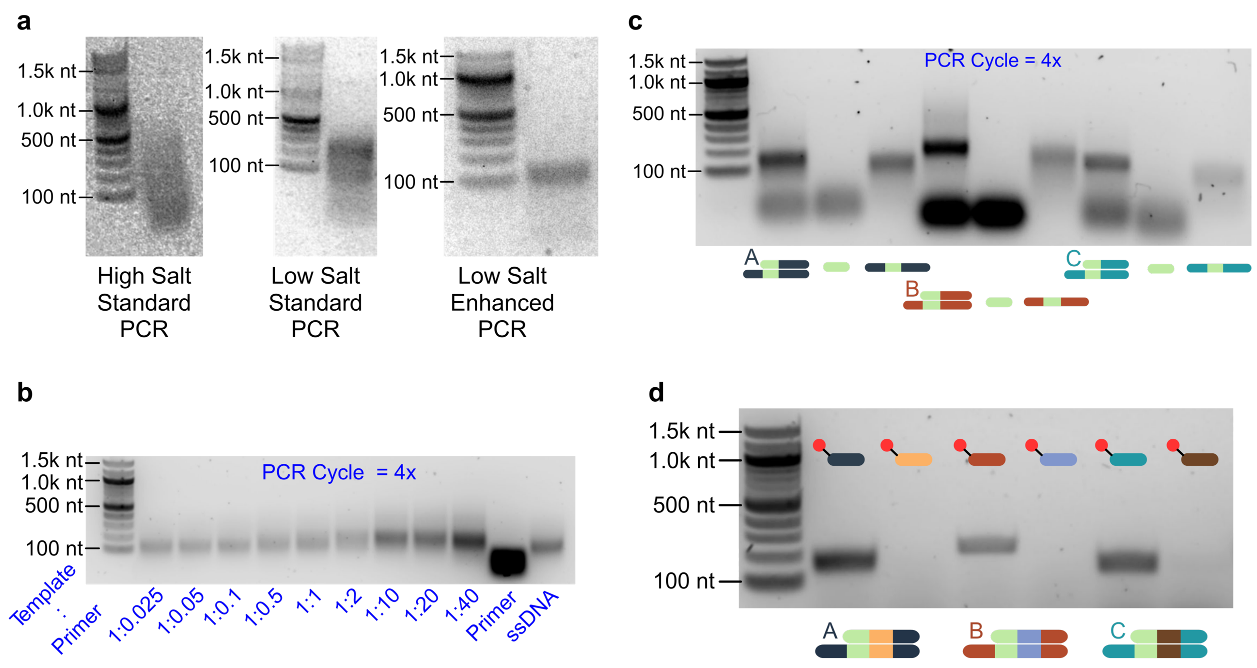

Supplementary Figure 1. Optimization of ss-dsDNA generation through single primer extension. **a** DNA gel electrophoresis. A low salt "enhanced" PCR buffer condition was identified that yielded a tight band corresponding to ss-dsDNA strands. Other PCR buffers resulted in multiple byproducts (buffer details described in Methods). **b** Increasing the amount of primer relative to ssDNA template in the primer extension process resulted in increasing amounts of ss-dsDNA strands generated, as seen in a shift upwards in size from ssDNA. A slight downward shift at ratios of 1:40 suggest production of excess ssDNA. **c** ss-dsDNA, primers, and ssDNA templates were run on a DNA gel to show their differences in band locations. **d** File separation oligos complementary to each ss-dsDNA's address or to the middle of the ss-dsDNA strand were mixed with samples after primer extension and then separated using magnetic beads. The absence of DNA when using oligos complementary to the middle of the strands indicate that the primer extension step was nearly complete in converting the ssDNA templates to ss-dsDNA strands, thus blocking 'non-specific' file separation. Gel images are representative of three independent experiments measured by RT-QPCR. Source data are provided as a Source Data file.

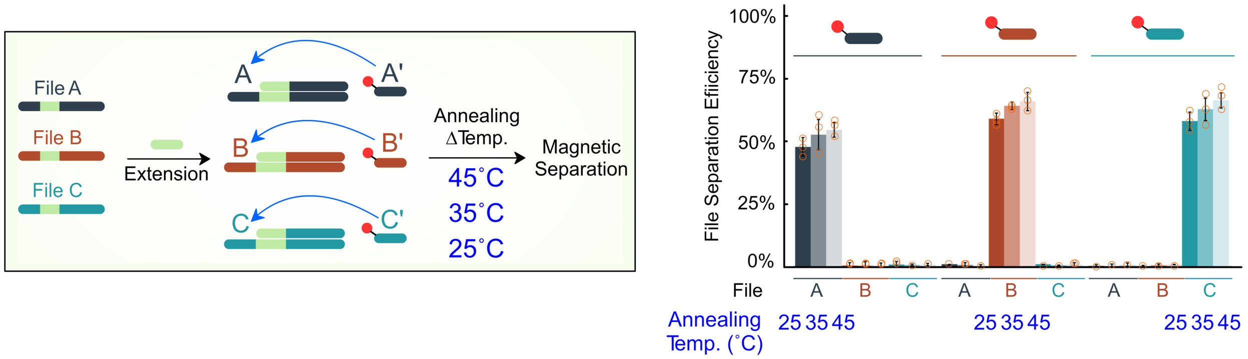

Supplementary Figure 2. File separation temperature does not appreciably affect separation efficiency from a three-file database.

After the three-file database was created, each file was bound by its corresponding biotin-linked oligo at 25, 35, or 45 ˚C and separated using magnetic beads (n=3 for each condition). The amounts of each file in the samples were quantified by qPCR. Each oligo separated its file specifically, and the annealing temperature did not appreciably affect the file separation efficiency, which was calculated as the amount of DNA separated relative to its original quantity in the database. Plotted values represent the arithmetic mean, and error bars represent the s.d., of three replicate file separations. Source data are provided as a Source Data file.

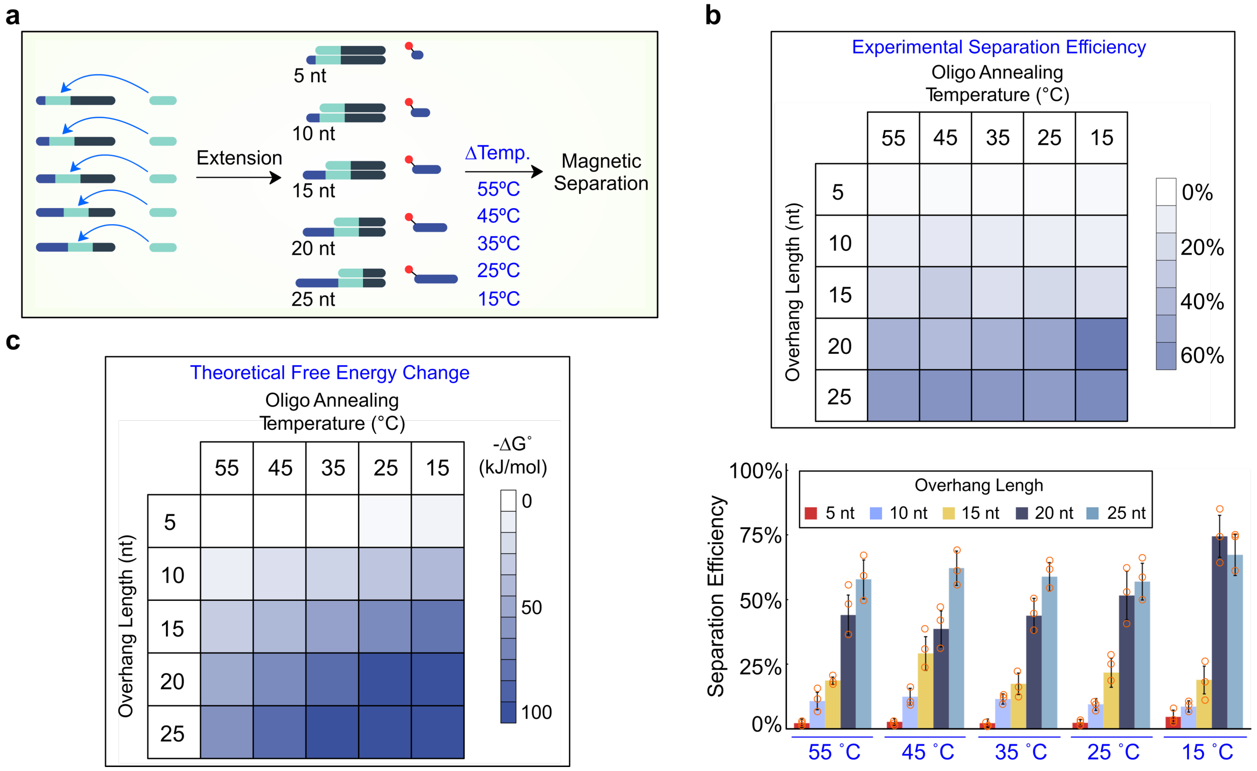

Supplementary Figure 3. Experimental and theoretical analyses map the dependency of file separation efficiency on oligo length and separation temperature.

a Five ss-dsDNA lengths were generated by single primer extension and then evaluated for their file separation efficiencies at five different temperatures (n=3 for each condition). **b** Experimental analysis of separation efficiency displayed as both heatmap and bar graph. The separation efficiency was calculated as the amount of file separated relative to its starting total quantity as measured by qPCR. **c** A theoretical analysis[1-3] of the change in Gibbs free energy at different oligo/ss-dsDNA lengths. Plotted values represent the arithmetic mean, and error bars represent the s.d., of three independent replicate file separations. Source data are provided as a Source Data file.

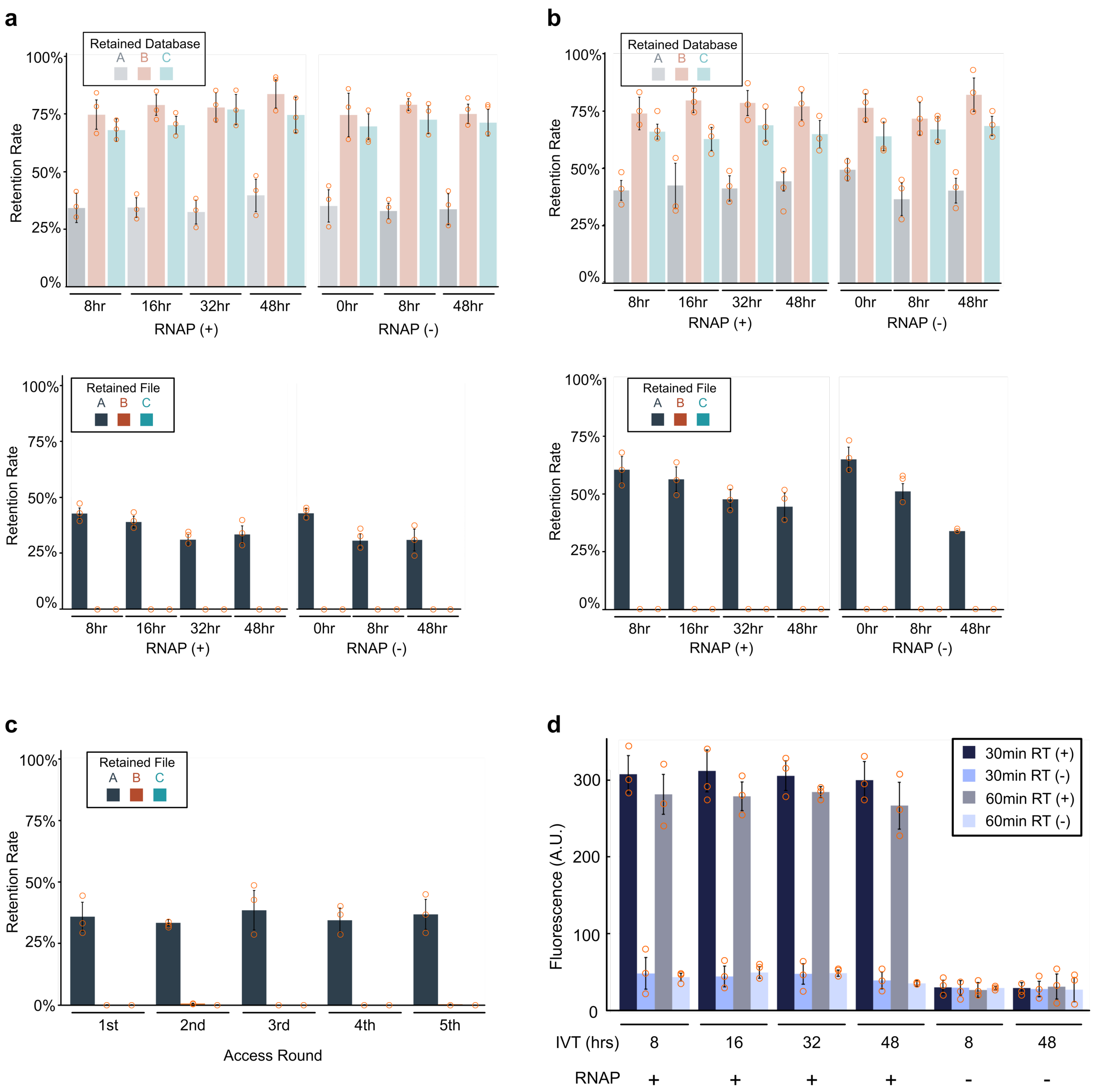

Supplementary Figure 4. IVT time but not the presence of RNA polymerase decreases the amount of Retained File.

a, b The presence/absence of RNA polymerease and IVT time did not affect the retention rate of the Retained Database (light shading) sample as these samples did not undergo IVT (n=3 for each condition). The presence/absence of RNA polymerase did not affect the retention rate of the Retained File (dark shading); however, the IVT time did (n=3 for each condition). **b** A re-annealing step to 45 ˚C was able to rescue some of this loss. Retention rate is the amount of DNA recovered relative to the starting amount of DNA in the original database. Error bars are standard deviations of three replicate IVTs. **c** The retention

rate of file A in the Retained File relative to the amount of file A in the database directly prior to each access round (n=3 for each condition). **d** cDNA generated from accessed file A was amplified by PCR, run on a DNA gel, and quantified by SYBR green fluorescence (n=3 for each condition). RNA polymerase (RNAP) was required to access file A. RT (reverse transcriptase). IVT (*in vitro* transcription). Plotted values represent the arithmetic mean, and error bars represent the s.d., of independent replicate file accesses. n=independent replicate file accesses. Source data are provided as a Source Data file.

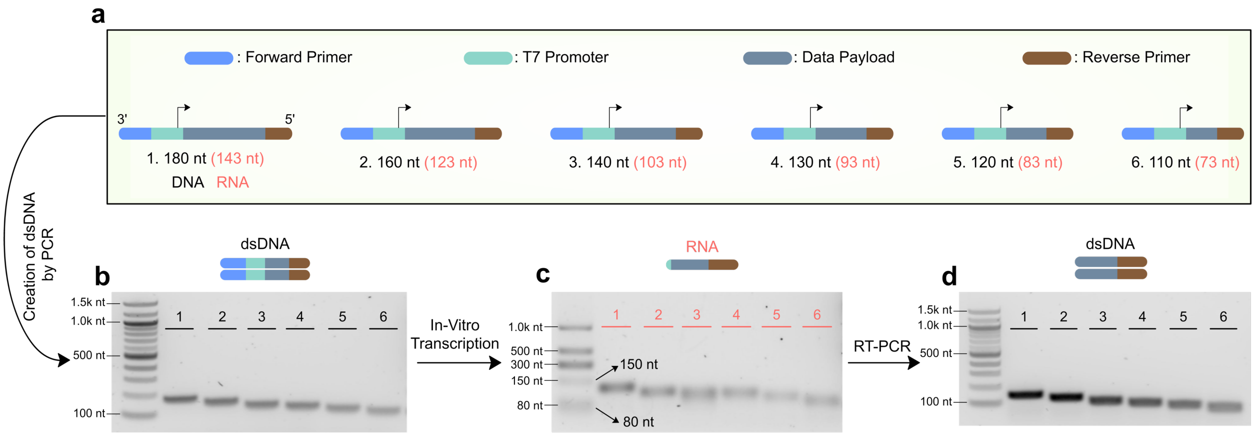

Supplementary Figure 5. T7-based transcription of dsDNA generates uniformly sized products.

a Six ssDNA oligos with different lengths were designed to generate six dsDNA templates with lengths of 180 bp, 160 bp, 140 bp, 130 bp, 120 bp and 110 bp, respectively. Each dsDNA comprised a consensus reverse primer binding sequence, T7 primer binding sequence, forward primer binding sequence, and a payload sequence with varying lengths. **b** These dsDNA templates were **c** *in-vitro* transcribed for 8 hours, followed by **d** RT-PCR. Product sizes were examined by agarose gel electrophoresis. Gel images are representative of three independent experiments measured by RT-QPCR. Source data are provided as a Source Data file.

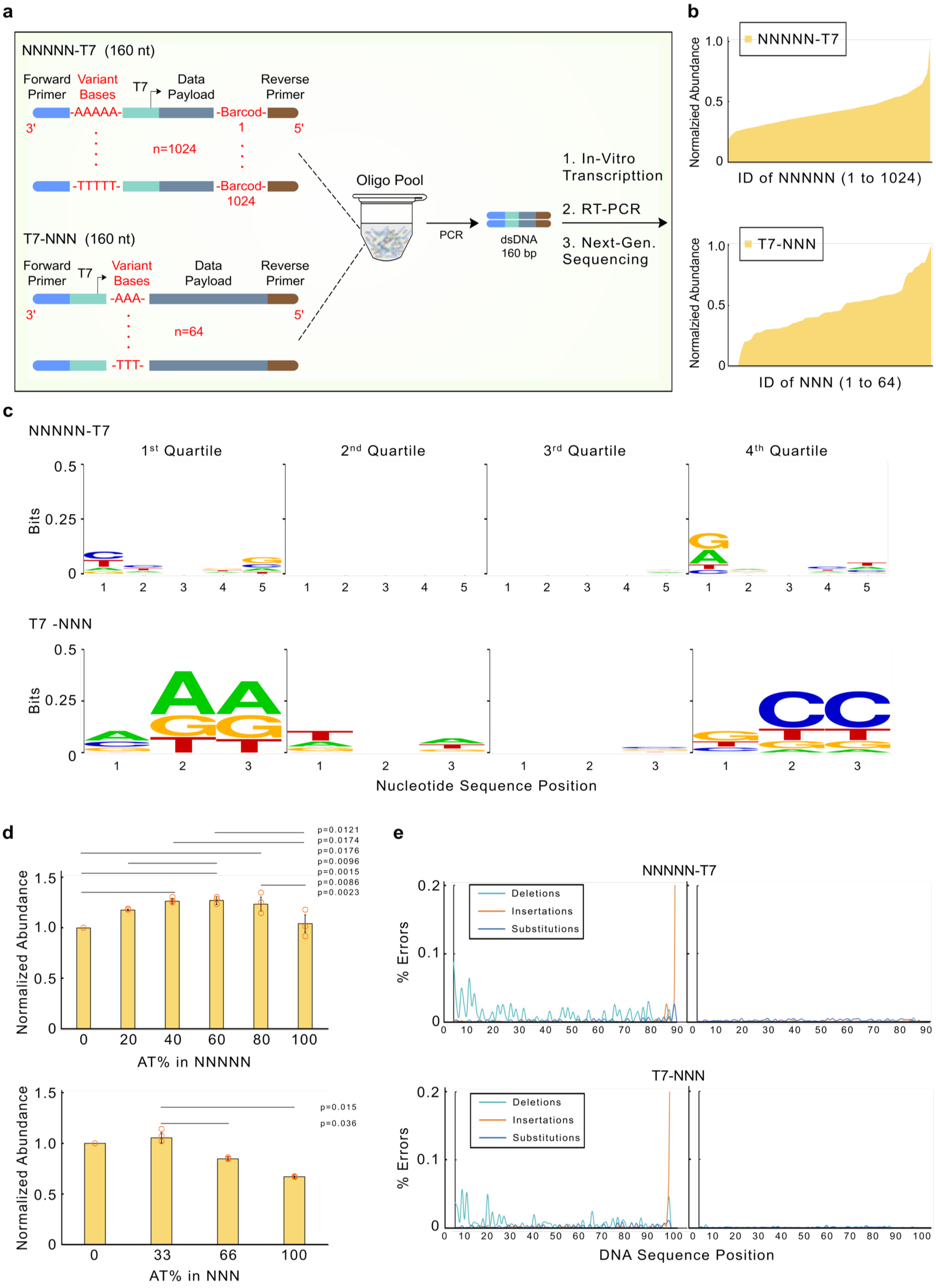
a
NNNNN-T7 (160 nt)
Forward Primer
Variant Bases
T7
Data Payload
Reverse Primer
-AAAAA-
-Barcod- 1
3'
5'
n=1024
-TTTTT-
-Barcod- 1024
T7-NNN (160 nt)
-AAA-
n=64
-TTT-
Oligo Pool
PCR
dsDNA 160 bp
1. In-Vitro Transcripttion
2. RT-PCR
3. Next-Gen. Sequencing
b
NNNNN-T7
Normalzied Abundance
ID of NNNNN (1 to 1024)
T7-NNN
ID of NNN (1 to 64)
c
NNNNN-T7
1st Quartile
2nd Quartile
3rd Quartile
4th Quartile
Bits
T7 -NNN
Nucleotide Sequence Position
d
p=0.0121
p=0.0174
p=0.0176
p=0.0096
p=0.0015
p=0.0086
p=0.0023
Normalized Abundance
AT% in NNNNN
p=0.015
p=0.036
AT% in NNN
e
NNNNN-T7
Deletions
Insertations
Substitutions
% Errors
T7-NNN
DNA Sequence Position

Supplementary Figure 6. T7-based transcription efficiency off of dsDNA templates can be controlled by surrounding sequences.

a An oligo pool that had 1088 distinct sequences was designed to generate dsDNA templates. The first 1024 sequences contained all possible combinations of nucleotides upstream of the promoter sequence (NNNNN-T7, where N is one of four DNA nucleotides), whereas the latter 64 sequences had all possible combinations of nucleotides downstream to the promoter region (T7-NNN). Each sequence contained a barcode to identify the sequence of the variant nucleotides. The template dsDNAs were processed with IVT for 8 hours, followed by RT-PCR and next-generation sequencing (n=3 for each condition). **b** Transcription efficiencies of both sequence designs were plotted by normalizing the read count of each transcribed strand to its abundance in the original library. The data was organized from lowest to highest normalized abundance for both designs. **c** The sequences were further divided into four quartiles based upon normalized transcript abundance and analyzed by the WebLogo tool[4]. **d** The normalized abundance of each sequence was organized by A/T percentage. P values were calculated using One-Way ANOVA with Tukey-Kramer post-hoc between each group and listed for statistical significance. NNNNN-T7: p values less than 0.05 for comparisons between 0%-80%, 40%-100% and 60%-100%; p values less than 0.01 for comparisons between 0%-40%, 0%-60%, 20%-60%, and 80%-100%. T7-NNN: p values less than 0.05 for comparisons between 33%-100%, 33%-66%. **e** The percent error for each DNA sequence position for the original database (left) and transcribed database (right). The error rate was calculated by dividing the number of errors of a given type occurring at a nucleotide position by the total number of reads for that sequence. Plo tted values represent the arithmetic mean, and error bars represent the s.d., of three independent IVT-RT-P CR-NGS samples. Source data are provided as a Source Data file.

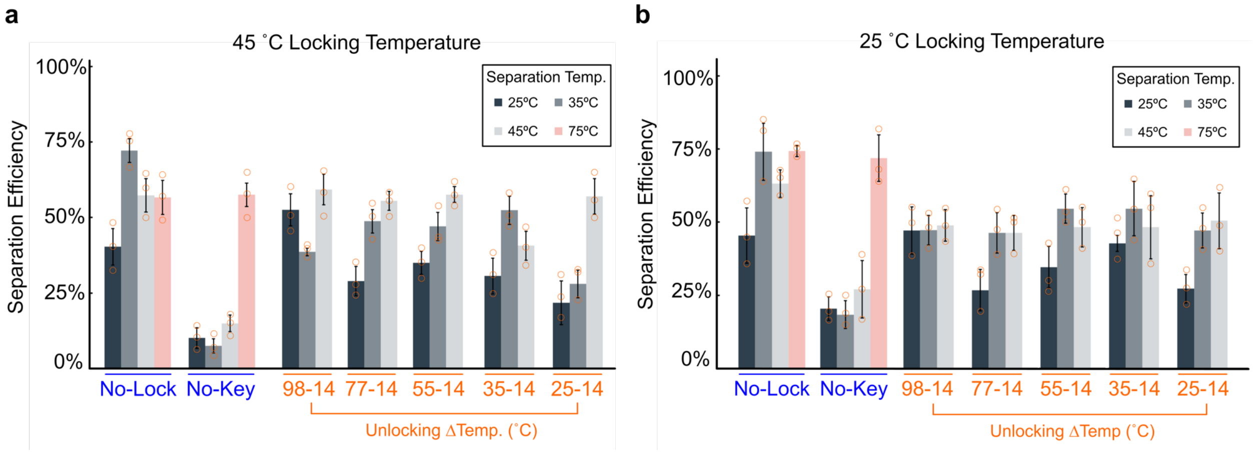

Supplementary Figure 7. Temperature influences the extent of locking.

File A was accessed by DORIS without locking, or following provision of a lock was accessed with or without subsequent unlocking by a key. The lock was added at **a** 45 °C or **b** 25 °C and then cooled to 14 °C. Oligo A' was added at different access temperatures of 25, 35, 45, or 75 ˚C for 2min, followed by a temperature drop of 1 ˚C/min to 25 °C (n=3 for each condition). Separation efficiency is the amount of file A recovered relative to its original quantity, as measured by qPCR. Plotted values represent the arithmetic mean, and error bars represent the s.d., of three replicate file separations. Source data are provided as a Source Data file.

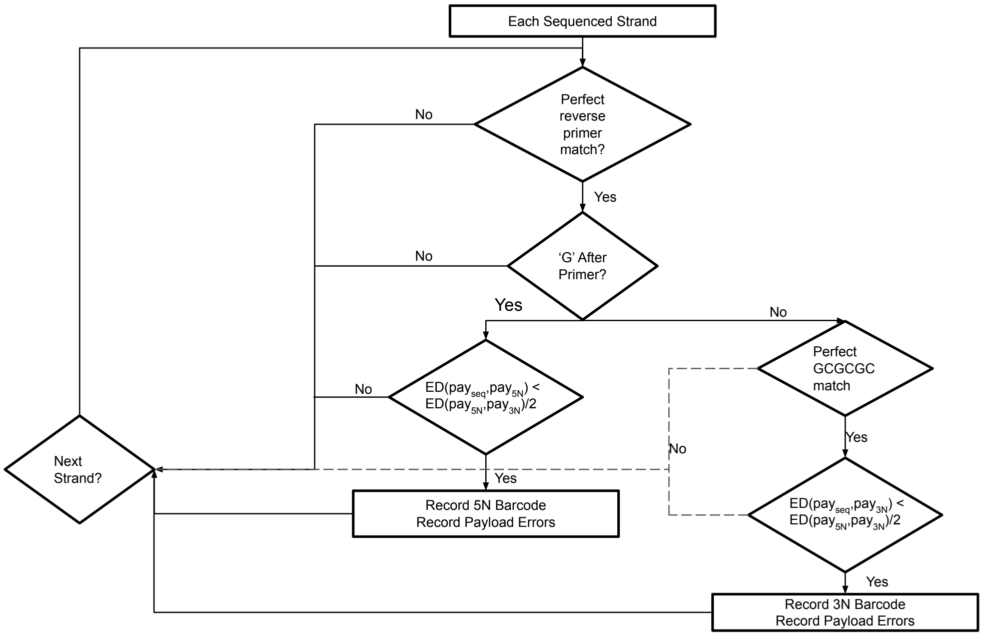

Supplementary Figure 8. **Flow chart to analyze and count the NGS sequencing samples for presence of NNNNN-T7 and T7-NNN strands**.

We counted the abundance of barcodes in a given sequencing sample by taking each sequenced strand and running through this flow chart. First, we searched for a perfect match of the reverse primer. If no match for the reverse primer was present in the strand, it was discarded and the next strand was considered. Next, we determined which type of strand it was, either NNNNN-T7 or T7-NNN. If the first base beyond the primer was *G,* the strand was classified as NNNNN-T7 (denoted as 5N in the flowchart) strand type. Otherwise it was assumed that the strand is of T7-NNN (denoted as 3N in the flowchart) type. Next we confirmed the classification by comparing the payload of the strand to the expected payload of the template using edit distance, denoted ED(*a*,*b*) where *a* and *b* were the sequences being compared. We used a heuristic test that worked empirically to confirm our classification. Namely, we expected the edit distance of the payload to the classified type to be less than half of ED($payload_{5N}$, $payload_{3N}$). If the heuristic test was confirmed, the strand was concluded to be 5N, else the strand was discarded. The same procedure was followed for 3N strands, except before performing the edit distance comparisons, a perfect match of a substrand of the T7 Promoter region was searched for (*GCGCGC*) in order to establish a reference point to subsequently read the 3N barcode. Whenever a barcode was recorded, the errors in the payload region were counted using the results from the edit distance calculation. These error counts were used for calculating the error rates for bases within the payload region.

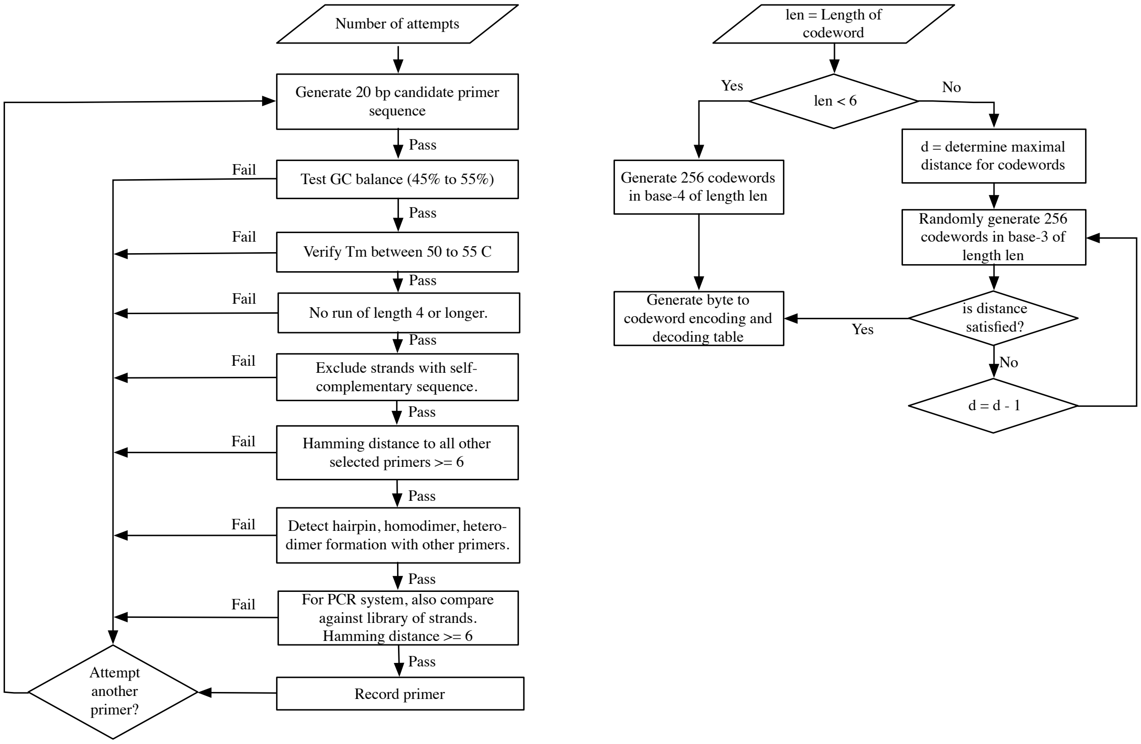

Supplementary Figure 9. Flowcharts for estimating total number of primers and for producing coding tables at varying density.

(Left) Flowchart for estimating viability of a primer implemented as a Python program. The overall process was a loop that repeated over some number of attempts to find a primer. Primer sequences were generated at random according to a uniform distribution of A, C, G, and T. Then, each primer was evaluated against several criteria. For estimating Tm (melting temperature), hairpins, and other dimer formation, we used the Primer3 software[5]. We required that all primers were at least a Hamming distance of 6 apart, and we required that they were at least a Hamming distance of 6 away from the target library. We approximated that requirement by comparing each primer to 1 MB of data that was randomly generated and encoded in each run of the program. The encoding of the library was also an input to the process that could be varied to evaluate the impact of coding density on primer selection. (**Right)** Flowchart for producing encoding and decoding tables of varying length. The data payload of strands, used to validate primers in the flowchart on the left, were created by encoding each byte one at a time as a codeword. The codeword tables at various lengths were created through a common algorithm. For length 4, all possible sequences were used, hence the creation is trivial. For length 5, we generated all possible sequences, and selected 256 of them at random. For lengths of 6 or longer, the process was different. We generated codes in base-3 (ternary) and select 256 of them, one for each possible single byte value. To ensure the codewords were different enough from each other, we first attempted to generate codes of maximal Hamming distance according to the Singleton bound.

If we did not succeed, we reduced the distance and tried again. The codeword tables created by the algorithm were manually verified to have a distance of 2 or more for all lengths greater than 6. For length 6 and higher, after encoding an entire strand, we used a rotating encoding to ensure no repetitions in the strand, neither within nor across codewords.

Supplementary Table 1. DNA oligomer sequences.

Sequences for the next-generation sequencing experiments are available at http://github.com/jamesmtuck/DORIS.

DNA Oligo	Sequence
ssDNA (File A)	CGTACGTACGTACGTCGACGGATGACAGCTCGCATCTACGAGCTCGAGATGACACAGAGTATCGCATCTACGACACAGTCTCTCGCGAGCTAGAGATGAGTGATCGAGCTCTGCTCGGCGCGCTATAGTGAGTCGTATTACGAGTGCAGAGCAGACTCAC
ssDNA (File A-2 for Truncated PCR)	CGTACGTACGTACGTCGACGGATGACAGCTCGCATCTACGAGCTCGAGATGACACAGAGTATCGCATCGAGTGCAGAGCAGACTCACAGCTAGAGATGAGTGATCGAGCTCTGCTCGGCGCG**C**TATAGTGAGTCGTATTACGAGTGCAGAGCAGACTCAC
ssDNA (File B)	CAGGTACGCAGTTAGCACTCCGTACGTACGTACGCAGCTAGCTCGATGAGTACTCTGCTCGATGAGTACTCTGCTCGACGAGATGAGACGAGTCTCTCGTAGACGAGAGCAGACTCAGTCATCGCGCTAGAGAGCATAGAGTCGTGATCTATGCTCAGCGCGCTATAGTGAGTCGTATTATCCGTAGTCATATTGCCACG
ssDNA (File C)	GGGAGTAATCCCCTTGGCGGTCGCGGGGGACAGCGCGTACGTGCGTTTAAGCGGTGCTAGAGCTGTCTACGACCAGCGCGCGCTATAGTGAGTCGTATTAGGATTCTCCAGGGCATCCGG
ssDNA (6 ss-dsDNA Templates) (180nt)	CAGGTACGCAGTTAGCACTCTACGCAGCTAGCTCGATGAGTACTCTGCTCGATGAGTACTCTGCTCGACGAGATGAGACGAGTCTCTCGTAGACGAGAGCAGACTCAGTCATCGCGCTAGAGAGCATAGAGTCGTGAGCGCGCTATAGTGAGTCGTATTATCCGTAGTCATATTGCCACG
ssDNA (6 ss-dsDNA Templates) (160nt)	CAGGTACGCAGTTAGCACTCTACTCTGCTCGATGAGTACTCTGCTCGACGAGATGAGACGAGTCTCTCGTAGACGAGAGCAGACTCAGTCATCGCGCTAGAGAGCATAGAGTCGTGAGCGCGCTATAGTGAGTCGTATTATCCGTAGTCATATTGCCACG
ssDNA (6 ss-dsDNA Templates) (140nt)	CAGGTACGCAGTTAGCACTCTAGCTCGACGAGATGAGACGAGTCTCTCGTAGACGAGAGCAGACTCAGTCATCGCGCTAGAGAGCATAGAGTCGTGAGCGCGCTATAGTGAGTCGTATTATCCGTAGTCATATTGCCACG
ssDNA (6 ss-dsDNA Templates) (130nt)	CAGGTACGCAGTTAGCACTCAGATGAGACGAGTCTCTCGTAGACGAGAGCAGACTCAGTCATCGCGCTAGAGAGCATAGAGTCGTGAGCGCGCTATAGTGAGTCGTATTATCCGTAGTCATATTGCCACG
ssDNA (6 ss-dsDNA Templates) (120nt)	CAGGTACGCAGTTAGCACTCAGTCTCTCGTAGACGAGAGCAGACTCAGTCATCGCGCTAGAGAGCATAGAGTCGTGAGCGCGCTATAGTGAGTCGTATTATCCGTAGTCATATTGCCACG
ssDNA (6 ss-dsDNA Templates) (110nt)	CAGGTACGCAGTTAGCACTCAGACGAGAGCAGACTCAGTCATCGCGCTAGAGAGCATAGAGTCGTGAGCGCGCTATAGTGAGTCGTATTATCCGTAGTCATATTGCCACG
Extension Oligo	TAATACGACTCACTATAGCGCGC

Separation Oligo A' for File A	GTGAGTCTGCTCTGCACTCG
Separation Oligo B' for File B	CGTGGCAATATGACTACGGA
Separation Oligo C' for File C	CCGGATGCCCTGGAGAATCC
Oligo B for Truncated PCR Product	CTACGACACAGTCTCTCGCG
PCR Forward Oligo for File A	GTGAGTCTGCTCTGCACTCG
PCR Forward Oligo for File B	CGTGGCAATATGACTACGGA
PCR Forward Oligo for File C	CCGGATGCCCTGGAGAATCC
PCR Reverse Oligo File A	CGTACGTACGTACGTCGACG
PCR Reverse Oligo File B	CAGGTACGCAGTTAGCACTC
PCR Reverse Oligo File C	GGGAGTAATCCCCTTGGCGGT
File A cDNA Forward Oligo	CGTACGTACGTACGTCGACG
File A cDNA Reverse Oligo	GAGCAGAGCTCGATCACTCA
File A Lock	CTCCATCAGAGTGATATGCCCAGCTTAGGTGAGTCTGCTCTGCACTCG
File A Key	CGAGTGCAGAGCAGACTCACCTAAGCTGGGCATATCACTCTGATGGAG
File A-> B Rename Oligo	TCCGTAGTCATATTGCCACGGTGAGTCTGCTCTGCACTCG
File A-> C Rename Oligo	GGATTCTCCAGGGCATCCGGGTGAGTCTGCTCTGCACTCG
File A Delete Oligo	GTGAGTCTGCTCTGCACTCG
6 ssDNA Templates Extension Oligo	TAATACGACTCACTATAGCGCGC
6 ssDNA Templates PCR Forward Oligo	CGTGGCAATATGACTACGGA

6 ssDNA Templates PCR Reverse Oligo	CAGGTACGCAGTTAGCACTC
6 ssDNA Templates cDNA Forward Oligo	GCTCACGACTCTATGCTCTC
6 ssDNA Templates cDNA Reverse Oligo	CAGGTACGCAGTTAGCACTC
Oligo Pool ss-dsDNA Extension Oligo	TAATACGACTCACTATAGCGCGC
Oligo Pool PCR Forward Oligo	CGTGGCAATATGACTACGGA
Oligo Pool PCR Reverse Oligo	CAGGTACGCAGTTAGCACTC
Oligo Pool PCR Forward Oligo after Poly A tailing	TTTTTTTTTTTTTTGCGCGC
Oligo Pool PCR Reverse Oligo after Poly A tailing	CAGGTACGCAGTTAGCACTC
Oligo Pool PCR Extension Forward Oligo (NNNNN-T7)	CACGATGAGCGACTTTTTTTTTTTTTTGCGCGC
Oligo Pool PCR Extension Reverse Oligo (NNNNN-T7)	GACTGAGTCACGTCAGGTACGCAGTTAGCACTC
Oligo Pool PCR Extension Forward Oligo (T7-NNN)	CACGATGAGCGACTTTTTTTTTTTTTTGCGCGC
Oligo Pool PCR Extension Reverse Oligo (T7-NNN)	GACTGAGTCACGTCAGGTACGCAGTTAGCACTC

Supplemental Methods

Error rates. The error analysis was performed based on the payload sequence of each strand. The Error rate was calculated by the number of errors of a given type seen at a base position divided by the total number of strands read for that sample. The total number of reads is the sum of all the barcode reads. The overall error rate per position across the whole dataset and the entire sequences for NNNNN-T7 and T7-NNN in both ss-dsDNA and dsDNA is listed as below:

**ss-dsDNA**	**Deletion**	**Insertion**	**Substitution**
NNNNN-T7	0.12% +/- 0.23%	0.04% +/- 0.20%	0.33% +/- 0.27%
T7-NNN	0.07% +/- 0.16%	0.03% +/- 0.06%	0.02% +/- 0.26%
**dsDNA**	**Deletion**	**Insertion**	**Substitution**
NNNNN-T7	0.03% +/- 0.13%	0.01% +/- 0.16%	0.04% +/- 0.15%
T7-NNN	0.04% +/- 0.07%	0.02% +/- 0.02%	0.17% +/- 0.05%

In ss-dsDNA experiments, deletion has an error rate of 0.12% per base in NNNNN-T7, but only 0.07% in T7-NNN. This is in comparison to the error rate induced by insertion, which generates 0.04% per base in NNNNN-T7 and 0.03% per base in T7-NNN. It is worth noting that among these error rates, the substitutions are most abundant with an error rate per base of 0.33% for NNNNN-T7 and 0.02% for T7-NNN in ss-dsDNA, and of 0.04% for NNNNN-T7 and 0.17% for T7-NNN. For ss-dsDNA case, it seems the error rates are higher in NNNNN-T7 than its in T7-NNN. Surprisingly, the overall error rate in dsDNA experiments are slightly lower than the ss-dsDNA case. However, it seems that the higher error rates are seen in T7-NNN, rather than in NNNNN-T7 sequence designs. Of note, a large proportion of errors are derived from the original database and therefore likely due to DNA synthesis errors. Error bars are standard deviations of three replicate IVT-RT-PCR-NGS samples.

Supplemental References

1. Sugimoto, N., Nakano, S. -i., Yoneyama, M. & Honda, K. -i. Improved Thermodynamic Parameters and Helix Initiation Factor to Predict Stability of DNA Duplexes. *Nucleic Acids Res.* **24**, 4501–4505 (1996).
2. Kibbe, W. A. OligoCalc: an online oligonucleotide properties calculator. *Nucleic Acids Res.* **35**, W43–W46 (2007).
3. Lomzov, A. A., Vorobjev, Y. N. & Pyshnyi, D. V. Evaluation of the Gibbs Free Energy Changes and Melting Temperatures of DNA/DNA Duplexes Using Hybridization Enthalpy Calculated by Molecular Dynamics Simulation. *J. Phys. Chem. B* **119**, 15221–15234 (2015).
4. Crooks, G. E. WebLogo: A Sequence Logo Generator. *Genome Res.* **14**, 1188–1190 (2004).
5. Untergasser A. et al. Primer3—new capabilities and interfaces. *Nucleic Acids Res*. **40**, 115 (2012).
